# Effectiveness of Digital Teaching Under Pandemic Conditions for the Donati Suture Technique

**DOI:** 10.7759/cureus.99359

**Published:** 2025-12-16

**Authors:** Eren Demir, Erik Wegner, Lars C Färber, Jochen Wollstaedter, Charlotte Arand, Thomas Ott, Erol Gercek, Sven O Dietz

**Affiliations:** 1 Department of Orthopaedics and Traumatology, Mainz University Medical Center, Mainz, DEU; 2 Department of Orthopaedics and Traumatology, Isar Klinikum Munich, Munich, DEU; 3 Department of Anaesthesiology, Mainz University Medical Center, Mainz, DEU

**Keywords:** digital teaching, donati-suture technique, instructional videos, medical education, surgical training

## Abstract

Background

The COVID-19 pandemic accelerated the expansion of digital teaching. However, both students and instructors reported challenges in effectively teaching practical skills under pandemic conditions. This study aimed to evaluate whether the Donati suture technique for wound closure can be effectively taught using instructional videos.

Methods

A total of 64 final-year medical students were randomly assigned to two groups (G1 and G2). At T0, G1 watched an instructional video on the Donati suture technique, while G2 performed self-practice exercises. At T1, both groups were tasked with closing a 5 cm wound within 10 minutes. After one week, G1 conducted self-practice exercises (T2) followed by a second wound closure task (T3), whereas G2 watched the video at T2 before performing the T3 task. Performance was evaluated using global and specific ratings.

Results

Both groups showed significant improvement from T1 to T3 (G1 p=0.012; G2 p=0.0002). At T3, there was no significant difference between groups. G2 demonstrated high variability at T1, which normalized at T3. G1 showed consistently low variability across all time points.

Conclusions

Digital instructional videos can effectively teach haptic skills such as the Donati suture technique. Learning outcomes are retained over time, and videos can help standardize skill acquisition among students with heterogeneous initial abilities.

## Introduction

In an increasingly complex clinical environment, teaching practical surgical skills faces multiple challenges, including limited resources, constraints on trainee work hours, rising operating room costs, and the adoption of minimally invasive techniques, all of which affect effective skill transmission [1]. These challenges were further intensified during the COVID-19 pandemic, which necessitated a rapid shift from traditional face-to-face instruction to digital teaching formats. The translation of practical surgical skills into digital formats presents unique obstacles for both educators and students [2].

Previous studies have demonstrated that foundational surgical skills can be effectively acquired through digital instructional materials. For instance, Shippey et al. showed that medical students could proficiently learn subcutaneous suturing techniques via instructional videos [1]. Despite the accelerated adoption of digital teaching methods in German university hospitals during the pandemic, feedback from students and educators highlighted persistent difficulties in teaching practical surgical content. This experience underscored the need for innovative approaches to practical skills education in medical training programs [3].

The present study aimed to evaluate whether the Donati suture technique for wound closure could be effectively taught to final-year medical students using instructional videos. We hypothesized that instructional videos could convey this surgical skill effectively and sustainably. Through this study, we sought to optimize the teaching of practical surgical skills at our institution, both under the constraints imposed by the COVID-19 pandemic and beyond.

## Materials and methods

The randomized controlled study was conducted during the summer term of 2021 at a university hospital. A total of 64 final-year medical students in their clinical year were prospectively and randomly assigned to two groups (G1 and G2).

An instructional video demonstrating the handling of surgical instruments and the Donati suture technique was produced by an experienced surgeon (SOD). The video had a duration of 10 minutes and illustrated both the proper handling of the required instruments and the stepwise execution of the Donati backstitch. The Donati technique was chosen because students had already learned the single stitch but not the backstitch technique in prior practical courses. At the time of study planning, the Donati suture was the standard backstitch technique in our department.

**Figure 1:**
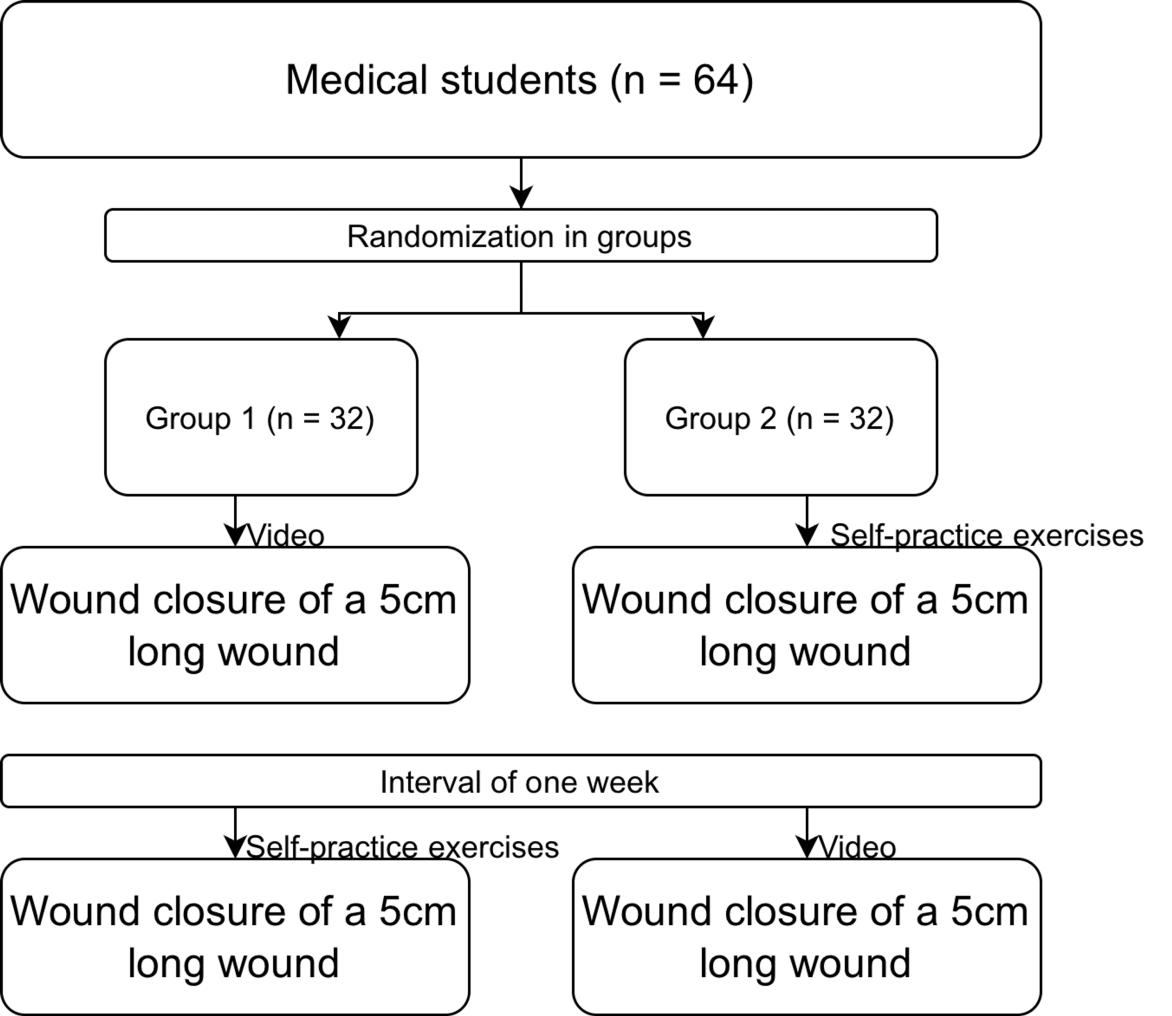
Study design

At baseline (T0), Group 1 (G1) watched the instructional video. Immediately afterward (T1), participants were asked to close a 5 cm wound using this technique within 10 minutes. Group 2 (G2) did not watch the video at T0; instead, they performed self-practice exercises and subsequently completed the same 5 cm wound closure under identical conditions at T1. After a one-week interval, both groups repeated the task: G2 watched the instructional video prior to performing the task (T2), while G1 engaged in self-practice exercises. Both groups then performed the wound closure again (T3). Figure 1 illustrates the study design and overall setup.

Student performance and application of the Donati suture technique were independently assessed by two orthopedic and trauma surgery specialists (SOD and LCF) using the validated assessment tool developed by Shippey et al. [1]. The assessment included a task-specific score (Table 1) and a global rating score (Table 2). For evaluation purposes, anonymized video recordings of students’ hands performing the sutures were made.

**Table 1:** Specific Score Cells marked with a dash (-) indicate that the observed outcome did not correspond exactly to a defined scoring category. Scores 1 and 5 represent the extremes (e.g., 1 = wound dehiscence, 5 = regular approximation of the wound edges), while scores 2–4 reflect intermediate outcomes that could not be clearly assigned to a single category. The dash signifies that the measurement fell somewhere within this intermediate range. The table was created by the authors, but its content is based on the scoring system described by Shippey et al. [1].

Correct orientation of the needle											
1			2	3	4		5		N/A		
Needle tilted			-	-	-		Needle punctures parallel to the edge of the wound		-		
Distance of the in/out stitches to the edge of the wound											
1		2		3		4	5				N/A
Insufficient, too far or too close		-		-		-	According to the needle diameter				-
Wound closure											
1	2			3		4		5		N/A	
Wound edges insufficiently adapted	-			-		-		Stitches in the same vertical plane		-	
Aesthetic result											
1	2			3		4		5		N/A	
Wound dehiscences	-			-		-		Regular approximation of the wound edges		-	

**Table 2:** Global Score Cells marked with a dash (-) indicate that the observed outcome did not correspond exactly to a defined scoring category. Scores 1 and 5 represent the extremes, while scores 2–4 reflect intermediate outcomes that could not be clearly assigned to a single category. The dash signifies that the measurement fell somewhere within this intermediate range. The table was created by the authors, but its content is based on the scoring system described by Shippey et al. [1]

Tissue managment									
1			2		3	4		5	N/A
Frequent tissue trauma due to unnecessary force or instrument use			-		Adequate tissue treatment, but occasional tissue trauma	-		Consistently good tissue managment	-
Instrument use									
1		2		3		4		5	N/A
Clumsy or inadequate handling of instruments		-		Competent use of instruments but sometimes clumsy		-		Constant skillful use of instruments	-
Time and movement									
1	2			3		4	5		N/A
Lots of unnecessary movements	-			Effective movements but sometimes unnecessary movements		-	Maximum efficiency		-
Wound closure fluid									
1	2			3		4	5		N/A
Often uncertainty about the next step	-			Adequate progress in wound closure		-	Excellent progress		-

Statistical analyses were performed using standard descriptive statistics, including mean ± standard deviation (SD) and range. Differences between time points were evaluated using paired t-tests for non-normal data. Statistical significance was set at p < 0.05. Analyses were performed using SAS software 9.4 (SAS Institute, Cary, North Carolina, USA). A difference of 2.1 points in the Global Rating Form and 3.4 points in the Task-Specific Form was considered significant. Improvements in scores for both groups were analyzed using paired t-tests with Bonferroni correction for multiple comparisons (two tests per group).

All procedures were performed in accordance with the Helsinki Declaration of 1964, and all patients provided verbal and written consent for participation prior to inclusion. The study was approved by the Ethics Committee of the State Medical Association Rheinland-Pfalz, approval number: 2021-15807. The trial was registered at clinicaltrials.gov NCT04842357.

## Results

**Figure 2:**
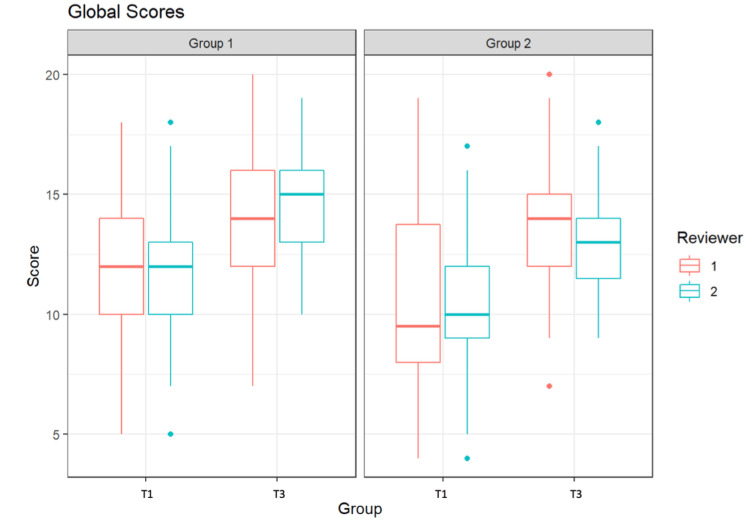
Global Scores of the students from Groups 1 and 2 by two independent reviewers at time point T1 and at time point T3

Figure 2 shows the assessment of students of G1 and G2 using the Global Score at T1 and T3 by two independent reviewers. A maximum score of 20 points could be achieved. The figure indicates that at T1, Group 1 was evaluated by the first reviewer with a median score of 12.0. The second reviewer assessed this group at the same time point with a score of 12.0. At time point T3, reviewer 1 rated the performance of G1 with a median of 14.0 points, and reviewer 2 with 15.0 points. Group 2 was assessed by the first reviewer at T1 with a median score of 9.5, and by the second reviewer with a score of 10.0. At time point T3, reviewer 1 rated the performance of G2 with a median of 14.0 points, and reviewer 2 with 13.0 points. 

**Figure 3:**
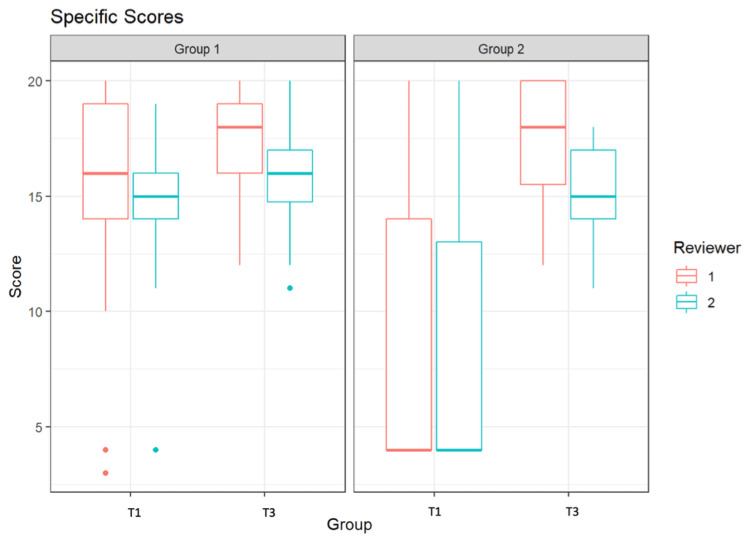
Specific Scores of the students from Groups 1 and 2 by two independent reviewers at time point T1 and at time point T3

Figure 3 displays the assessment of students by the two reviewers using the Specific Score. Here, a maximum score of 20 points could also be achieved. At T1, Group 1 was assessed by the first reviewer with a median score of 16.0, and by the second reviewer with a score of 15.0. At T3, Group 1 received a median score of 18.0 from the first reviewer and a score of 16 from the second reviewer.

At T1 and T3, reviewer 1 rated the performance of G2 with a median score of 4 and 18, respectively, and reviewer 2 with a median score of 4 and 15, respectively. 

**Figure 4:**
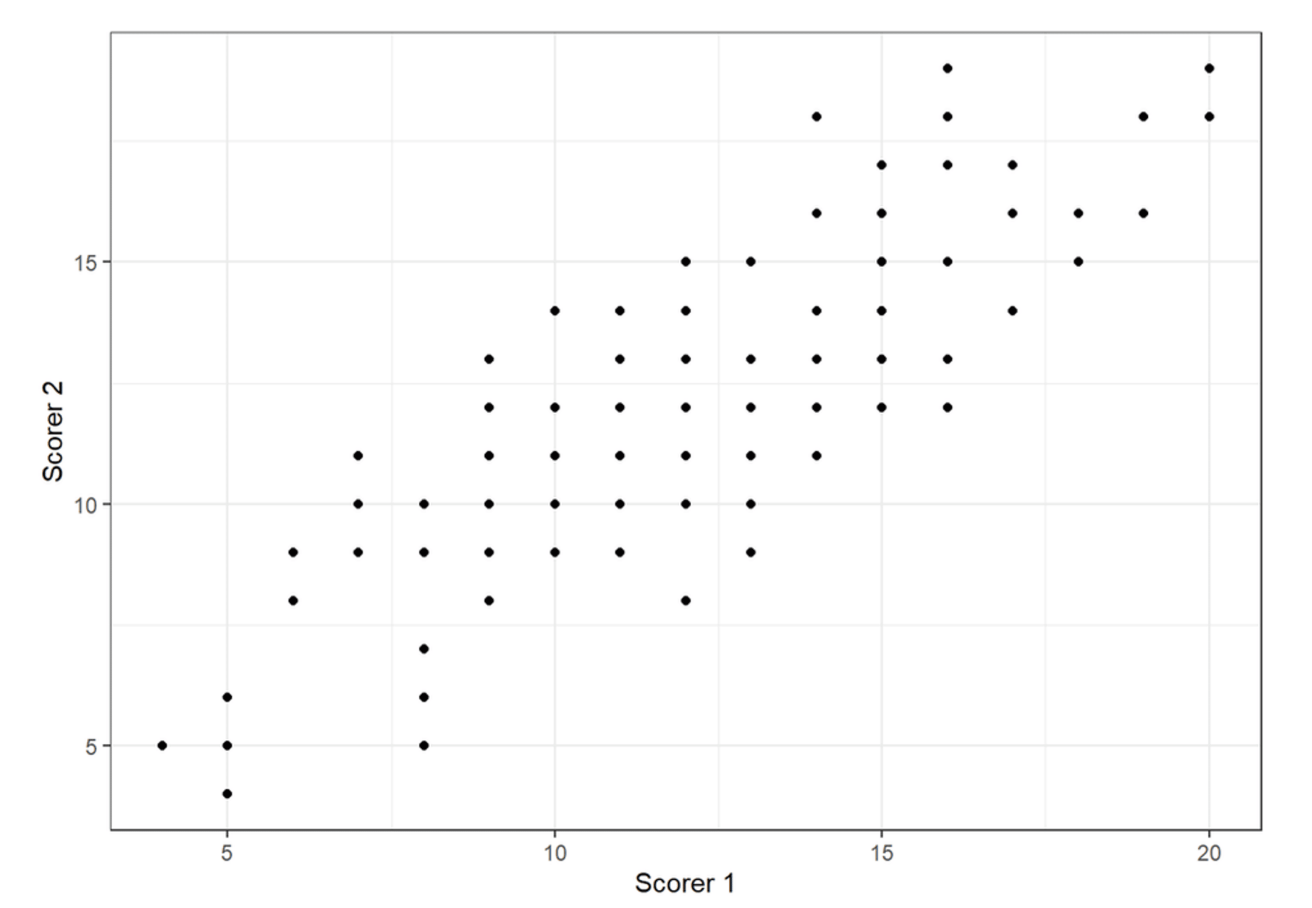
Inter-rater reliability: Scatter plot of all reviewers by reviewer and correlations

Figure 4 shows the inter-rater reliability of the two independent reviewers. The results of the conducted study demonstrated a good Intra-Class Correlation (ICC) of 0.83 among the evaluators.

**Figure 5:**
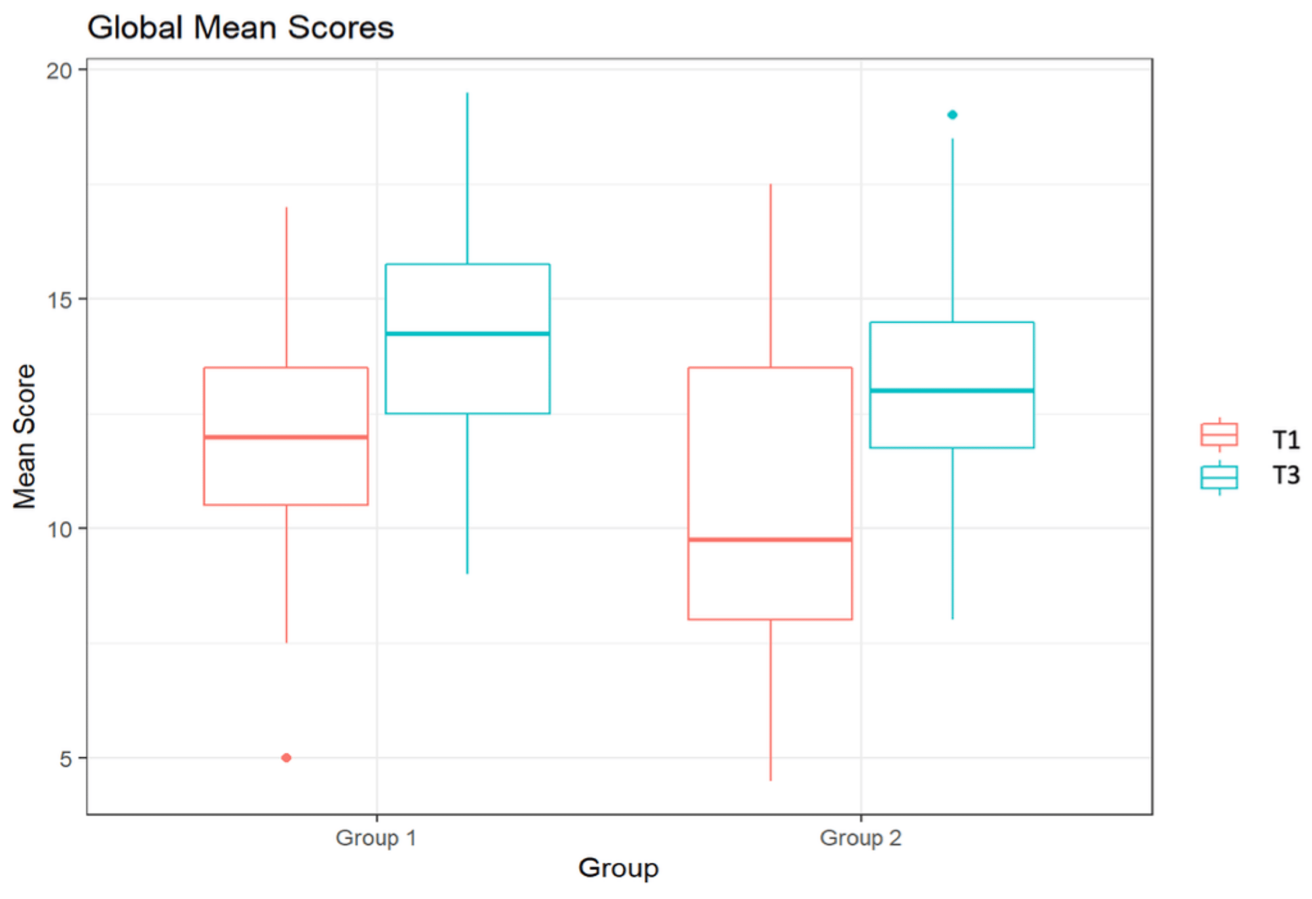
Global Mean Scores of the students from groups 1 and 2 across reviewers at time point T1 and at time point T3

Figure 5 illustrates the cross-reviewer assessment using the Global Score. It shows that at T1, Group 1 had a median score of 12.00, while the median score for this group at T3 was 14.25. Group 2 had a median score of 9.75 at T1 and a median score of 13.00 at T3.

**Figure 6:**
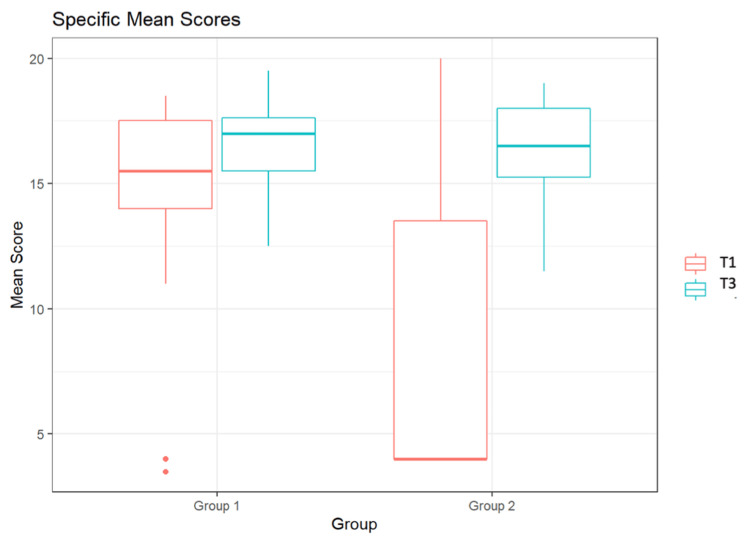
Specific Mean Scores of the students from Groups 1 and 2 across reviewers at time point T1 and at time point T3

Figure 6 displays the cross-reviewer assessment of students using the Specific Score. At T1, Group 1 had a median score of 15.5, while the median score at T3 was 17.0. Group 2 had a median score of 4.0 at T1 and a median score of 16.5 at T3.

Both groups exhibited a significant improvement in both "global" and "specific rating" between T1 and T3 (Group 1 p=0.012; Group 2 p=0.0002). The results remained significant across evaluators. There was no significant difference between the results of the two groups at T3. On average, students in Group 1 improved by 1.9 points, while those in Group 2 improved by 8.6 points. The maximum score attainable was 40 points. It is noteworthy that the results of Group 2 showed an extremely wide dispersion at T1, which was no longer present at T3. In Group 1, there was no wide dispersion observed at either T1 or T3.

## Discussion

This study demonstrated that practical surgical skills can be effectively and sustainably taught through instructional videos. The results from Group 2 indicated that a broad range of skill levels among students could be significantly elevated to a uniform level using a digital instructional video. Furthermore, Group 1 showed that this learning effect persists over time. Our data clearly show that the Donati suture technique can be successfully and sustainably learned via an instructional video. Especially during the COVID-19 pandemic and the related restrictions, this video-based teaching format provided a crucial alternative to traditional surgical skills instruction. The results suggest that this learning method remains valuable beyond the pandemic, particularly considering increasing economic pressures and limited resources for in-person teaching in hospitals.

The benefits of video-based surgical skills training are well documented. Shippey et al. highlighted that videos create focused learning opportunities by allowing students to review specific steps and address difficulties through targeted segments. Additionally, videos provide a visual demonstration of the task's final product, enabling repeated viewing [1]. Literature also indicates that video-based learning significantly improves retention: learners retain about 10-15% of what they read, 10-20% of what they hear, and 20-30% of what they see, with combined audio-visual materials boosting retention to 40-50% [4].

The literature remains inconclusive on whether additional in-person instruction further enhances learning outcomes. Rogers et al. reported better performance in knot-tying skills when expert feedback supplemented instructional videos [5], while Nousianen et al. found comparable outcomes between video-only and video-plus-expert instruction groups [6]. Notably, these studies did not evaluate retention. Comparing digital and personal instruction is complicated by the time-intensive nature of in-person teaching and potential instructor-related bias. Youssef et al. (2022) emphasized that video-based instruction standardizes teaching materials, creating uniform learning content. The *New England Journal of Medicine *(NEJM) surgical video series exemplifies this approach; Saun et al. demonstrated its superiority over didactic methods for chest tube insertion, likely due to reduced human bias and variability [7,8]. Such bias is relevant when evaluating surgical skills [9]. Unlike Shippey et al., who used a single expert evaluator, our study minimized bias by involving two independent experts, with high inter-rater reliability (ICC = 0.83) [1].

Deliberate practice is key to improving medical skills, similar to athletic training, as emphasized by Ericsson et al. [10]. Summers et al. found that digital learning led to better retention of basic surgical skills than instructor-led training, though their study allowed participants free practice during a month-long interval [11]. Our study featured a one-week interval without additional practice, raising questions about the effect of self-practice during retention periods.

A limitation of our study is the absence of a direct comparison between in-person and digital teaching. Group 1 watched the instructional video first, while Group 2 engaged only in self-practice initially, without personal demonstration. Thus, we cannot conclusively compare digital to traditional instruction quality.

Moreover, most literature focuses on simple surgical skills. The reproducibility of video-based learning for complex skills warrants further investigation. Wong et al. stress the need for multimodal approaches beyond traditional operating room teaching [12], often involving simulation training. Thomaschewski et al. conducted a randomized study showing that repeated viewing of standardized laparoscopic video tutorials improved technical skill precision and learning curves [13]. However, these videos complemented rather than replaced traditional training, so the question of video-only learning for complex skills remains open.

Another limitation is our use of a simple video format. Recent studies indicate that virtual reality training outperforms video-based methods for both simple and complex skills. Lohre et al. showed that immersive virtual reality led to higher Objective Structured Assessment of Technical Skills (OSATS) scores in reverse shoulder arthroplasty training compared to video learning [14]. Similarly, Yoganathan et al. reported better outcomes in virtual reality groups for basic surgical skills acquisition versus video-only groups [15].

Furthermore, the follow-up period in our study was limited to only one week. Although our results indicate short-term retention, this timeframe does not allow conclusions about long-term skill preservation. Future studies should incorporate extended follow-up intervals to assess long-term outcomes

## Conclusions

Our results demonstrate that simple surgical skills can be successfully and sustainably learned through instructional videos. The benefits of such instructional videos in medical education in a changing hospital society have been highlighted. The question of whether instructional videos are superior to in-person teaching and to what extent additive learning effects can be achieved through instructional videos remains unanswered by our data. Similarly, the question of whether the results of this study can be applied to more complex surgical skills remains unanswered. Digital teaching materials represent an important tool for future education. Further data will be needed for optimal handling of this type of teaching.
